# Manipulation of dipeptidylpeptidase 10 in mouse and human *in vivo* and *in vitro* models indicates a protective role in asthma

**DOI:** 10.1242/dmm.031369

**Published:** 2018-01-01

**Authors:** Youming Zhang, Thanushiyan Poobalasingam, Laura L. Yates, Simone A. Walker, Martin S. Taylor, Lauren Chessum, Jackie Harrison, Loukia Tsaprouni, Ian M. Adcock, Clare M. Lloyd, William O. Cookson, Miriam F. Moffatt, Charlotte H. Dean

**Affiliations:** 1 Genomics Medicine Section, National Heart and Lung Institute, Imperial College London, London, SW3 6LY, UK; 2 Inflammation, Repair and Development Section, National Heart and Lung Institute, Imperial College London, London, SW7 2AZ, UK; 3 Wellcome Trust Centre for Human Genetics, University of Oxford, Oxford, OX3, 7BN; 4 MRC Harwell Institute, Oxfordshire, OX11 0RD, UK; 5 Airway Disease Section, National Heart and Lung Institute, Imperial College London, London, SW3 6LY, UK

**Keywords:** DPP10, Point mutation, IgE, Airway resistance, Asthma

## Abstract

We previously identified dipeptidylpeptidase 10 (*DPP10*) on chromosome 2 as a human asthma susceptibility gene, through positional cloning. Initial association results were confirmed in many subsequent association studies but the functional role of DPP10 in asthma remains unclear. Using the MRC Harwell N-ethyl-N-nitrosourea (ENU) DNA archive, we identified a point mutation in *Dpp10* that caused an amino acid change from valine to aspartic acid in the β-propeller region of the protein. Mice carrying this point mutation were recovered and a congenic line was established (*Dpp10^145D^*). Macroscopic examination and lung histology revealed no significant differences between wild-type and *Dpp10^145D/145D^* mice. However, after house dust mite (HDM) treatment, *Dpp10* mutant mice showed significantly increased airway resistance in response to 100 mg/ml methacholine. Total serum IgE levels and bronchoalveolar lavage (BAL) eosinophil counts were significantly higher in homozygotes than in control mice after HDM treatment. DPP10 protein is present in airway epithelial cells and altered expression is observed in both tissue from asthmatic patients and in mice following HDM challenge. Moreover, knockdown of *DPP10* in human airway epithelial cells results in altered cytokine responses. These results show that a *Dpp10* point mutation leads to increased airway responsiveness following allergen challenge and provide biological evidence to support previous findings from human genetic studies.

This article has an associated First Person interview with the first author of the paper.

## Abstract (precis)

**Summary:** Here, we show a novel mouse model carrying a point mutation in dipeptidylpeptidase 10 (Dpp10). Our data provide evidence that DPP10 might play a protective role in asthma.

## INTRODUCTION

Asthma is characterized by intermittent inflammation of the large airways in the lungs with symptoms of wheeze and shortness of breath. The disease is caused by a combination of genetic and environmental factors. Previously, we have established that polymorphisms in *DPP10* on human chromosome 2 were associated with asthma traits through positional cloning (Allen et al., 2003). These associations have since been replicated in different ethnic populations worldwide (Wu et al., 2010; Blakey et al., 2009; Zhou et al., 2009; Gao et al., 2010; Mathias et al., 2010). To date, *DPP10* is the only gene found to show asthma association by both positional cloning and genome-wide association studies (GWAS) (Mathias et al., 2010). *DPP10* encodes a single-pass type II membrane protein that is a member of the S9B family in clan SC of the serine proteases. It has no detectable protease activity in mammals, owing to the absence of a conserved serine residue normally present in the catalytic domain of these proteases (Qi et al., 2003). Instead, DPP10 has been shown to bind specific voltage-gated potassium channels, altering their expression and biophysical properties (Jerng et al., 2004). Although DPP10 has been associated with asthma in both genome-wide and wet-laboratory studies (Allen et al., 2003; Schade et al., 2008; Kim et al., 2015), its functional role in asthma is almost completely unknown, due in large part to the absence of any genetic models.

Mouse mutants are powerful experimental tools for the study of complex diseases, such as asthma. They have been of great benefit for mapping quantitative traits (Zhang et al., 1999; De Sanctis et al., 1995), and serve as experimental tools to dissect the functional roles of genes *in vivo* (Zhang et al., 2014). *Dpp10^145D^* mice were generated using N-ethyl-N-nitrosourea (ENU)-induced mutagenesis of the mouse genome, which is a conventional method employed for functional analysis (Yates et al., 2009).

To investigate the function of DPP10 *in vivo*, we obtained full-length mouse *Dpp10* complementary DNA (cDNA). We then sequenced four of the 26 exons of *Dpp10* in 3840 mouse DNA samples from the UK MRC Harwell archive of ENU-mutagenized F1 DNA samples. The four exons chosen for sequencing include the regions encoding the transmembrane and β-propeller domains of *Dpp10*, both of which are thought to be required for proper protein function (Allen et al., 2003). Here, we report the establishment of a novel mouse mutant carrying a DPP10 point mutation and its effects on experimental asthma.

## RESULTS

### Mouse *Dpp10* genomic structure

We identified the full-length mouse cDNA of *Dpp10* and aligned this with available information on *Dpp10*. Mouse genomic *Dpp10* is located on chromosome 1 and has 26 exons encoding 796 amino acids. The key domains found within the sequence are a transmembrane domain and a β-propeller region (Fig. S1). We therefore screened the ENU DNA archive for mutations within exons encoding either of these domains.

### Identifying and validating ENU mutations in *Dpp10*

**Fig. 1.**
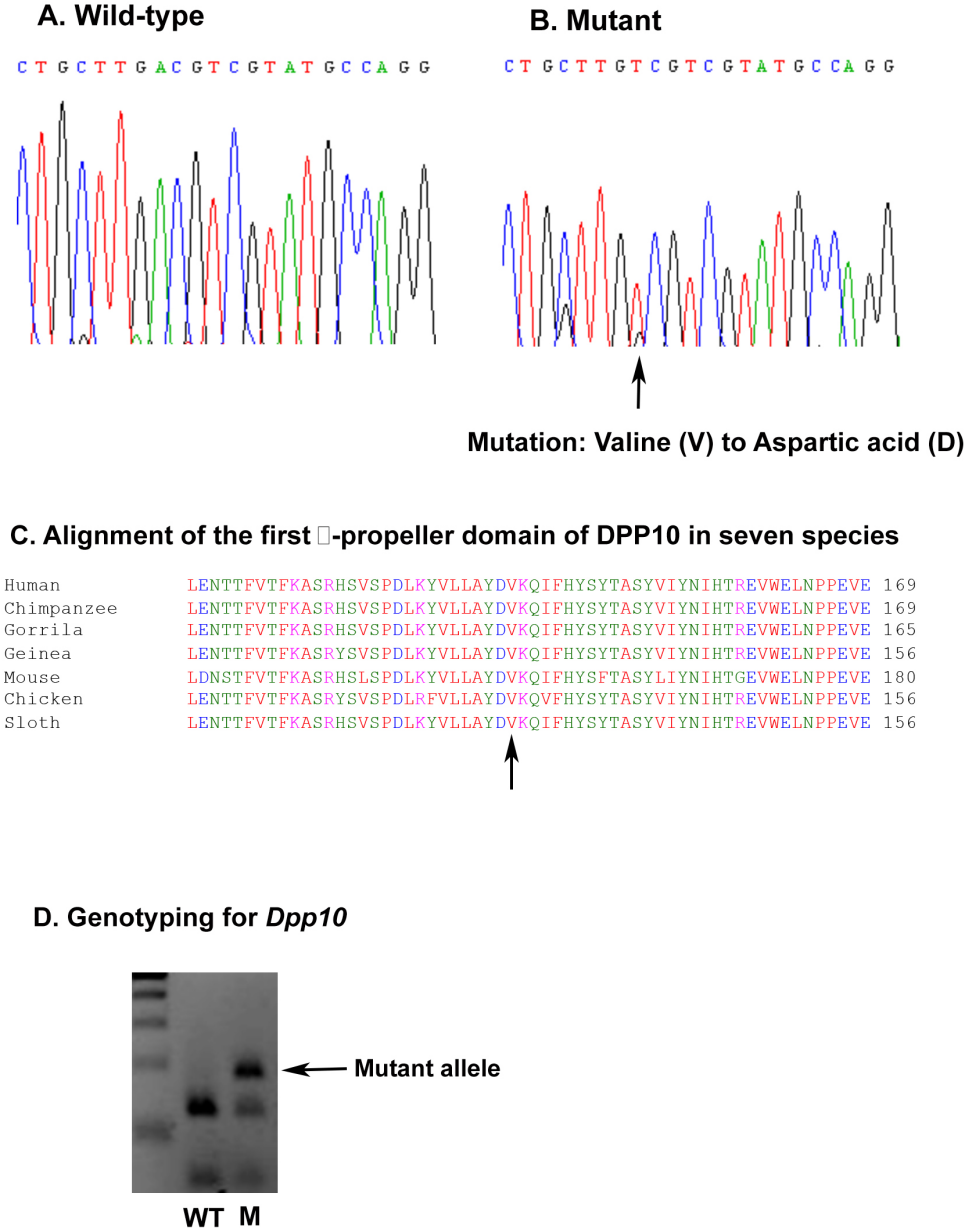
**The *Dpp10* mutation and genotyping in mice.** (A) Wild-type sequence for *Dpp10*. (B) Mutant sequence of *Dpp10*. (C) Alignment of the sequences containing the first β-propeller of DPP10 across seven species. (D) Gel comparing the genotyping results from wild-type (left lane) and heterozygous (right lane) *Dpp^145D^* littermates. Using forward 5′-AGTCTTGTCTTTACCACA-3′ and reverse 5′-AAGCCTCCAGACACTCAC-3′ primers, PCR generated a 194 bp product. Restriction enzyme digest with HpyCH4IV cut the wild-type allele at ACGT, producing two bands of 127 bp and 67 bp, whilst the mutant allele remained uncut (194 bp). WT, wild-type mouse, M, mutant mouse.

From this initial screening, we identified three mutations in 3480 DNA samples. The only nonsynonymous mutation found caused an amino acid change from valine to aspartic acid in exon 5 (Fig. 1A,B). The valine, or at least a hydrophobic residue at this position, is well conserved in DPP10 and DPP6 orthologous sequences throughout vertebrates (Fig. 1C). The wider family of DPPIV domain-containing proteins do not generally conserve this position, but aspartic acid (D) has not been found in any of the proteins defined by the Protein FAMilies database of alignments and Hidden Markov Models (PFAM) profile (Sonnhammer et al., 1998). The mutated residue is on the surface of the protein at the entrance to a pocket in the centre of the β-propeller region. A surface-exposed hydrophobic residue in such a position could well be involved in determining substrate specificity and therefore a mutation in this position is highly likely to have some functional impact on DPP10.

### Recovery and maintenance of *Dpp10* mutant mice

To determine whether the mutation identified would have an impact on DPP10 function *in vivo*, we established a mouse line carrying this mutation, using the corresponding F1 male sperm sample from the Harwell DNA archive (Yates et al., 2009). *In vitro* fertilization (IVF) with the mutagenized sperm was performed using C3H embryos to facilitate genotyping of the mutation that had been induced in Balb/C mice. We established a genotyping strategy using an enzymatic diagnostic digest with HpyCH4IV, followed by PCR, to distinguish mutant mice from wild-type littermates (Fig. 1D). Seven offspring were obtained, six of which contained the mutant allele, indicating that a single copy of the mutation did not affect mouse viability or fertility. We subsequently established a congenic *Dpp10* line by backcrossing these mice to CH3 for 10 generations, thereby ensuring that the line did not contain any additional ENU mutations. *Dpp10* heterozygotes were intercrossed and the genotypes of their offspring were analysed at E18.5. Normal Mendelian ratios of homozygous, heterozygous and wild-type embryos were found, indicating no prenatal mortality and this was confirmed by Chi-squared analysis. Postnatally, *Dpp10^145D^* homozygotes and heterozygotes were also recovered in expected numbers and these were morphologically indistinguishable from wild-type littermates. All subsequent experiments were conducted using either homozygous *Dpp10* mice or their wild-type littermates.

### Histological examination of *Dpp10* mutant mouse lungs

**Fig. 2.**
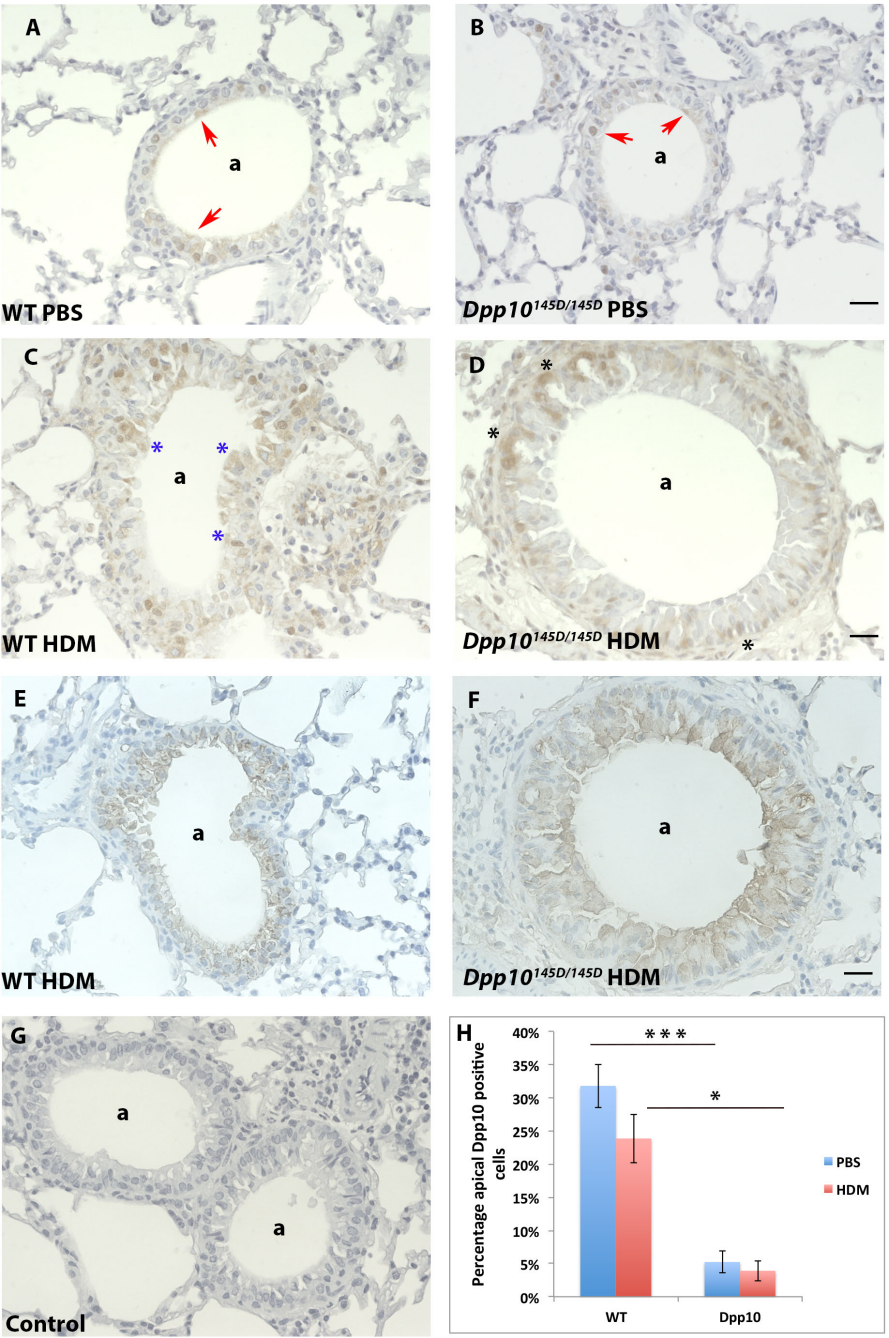
**Dpp10 localization in mouse airways.** (A-D) Dpp10-positive staining is present in some airway epithelial cells in both wild-type (A) and homozygous (B) lungs; red arrows indicate examples of positively stained cells. Treatment with house dust mite (HDM) resulted in increased DPP10 staining in both genotypes (C,D). In wild-type mice, Dpp10-positive staining was frequently present at the apical surface of airway epithelial cells (blue asterisks) (C). In *Dpp10^145D^* homozygotes, few cells with apical Dpp10 staining were present. By contrast, strong staining was frequently observed more basally (black asterisks) (D). (E,F) Many cells in both WT (E) and *Dpp10^145D^* homozygous airways (F) are positive for the club cell marker CC10. (G) Haematoxylin-stained control airways with primary antibody omitted. (H) Quantification of cells with apical Dpp10 staining from eight airways in *n*=3 mice per group. Data are mean±s.e.m., Student's *t*-test, ***P<0.001 for WT versus *Dpp10^145D^* PBS-treated mice and *P<0.05 for WT versus *Dpp10^145D^* HDM-treated mice. Scale bars: 12.5 µM. a, airway lumen.

*Dpp10^145D^* homozygotes survived to term and histological analysis did not reveal any visible differences in adult lung architecture (Fig. S2). We investigated the localization of Dpp10 protein in both wild-type and homozygous *Dpp10* mutant lungs by immunostaining. In phosphate-buffered saline (PBS)-treated wild-type and *Dpp10^145D/145D^* lungs, we observed a subset of airway epithelial cells with Dpp10-positive staining (Fig. 2A,B). Dpp10 staining was much more visible in the airways of both wild-type and mutant mice treated with HDM than in PBS-treated mice (Fig. 2C,D). Staining of sections from the same samples shown in Fig. 2C and D with club cell-10 (CC10), a club cell marker, highlights differences in the patterns of Dpp10 and CC10 staining (Fig. 2E,F). The discrepancy is particularly striking in *Dpp10* homozygous airways (Fig. 2D versus F). More detailed comparison of Dpp10 localization in wild-type and *Dpp10^145D^* airway cells showed that in wild-type airways, staining was frequently observed at the apical surface of cells, whereas in mutant airways, cells with apical Dpp10 staining were much rarer (*P*<0.05, Fig. 2F,H). A control section where the primary antibody was omitted shows no DAB staining (Fig. 2G). These data indicate that the point mutation in *Dpp10^145D^* leads to altered protein localization rather than complete loss of protein. Further analysis of the lungs showed no visible differences in the cell-type specific markers pro-surfactant protein-C, CC10, smooth muscle actin (SMA) or aquaporin-5 between *Dpp10* homozygotes and wild-type mice (Fig. S3), indicating that the *Dpp10* mutation does not affect cell differentiation.

### The response of *Dpp10* mutant mice to house dust mite challenge

To determine whether the *Dpp10^145D^* mutation had a functional impact and, in particular, whether the mutation might modify airway hyper-responsiveness (AHR), we dosed wild-type (*n*=9) and *Dpp10* homozygous (*n*=10) mice with 25 µg house dust mite extract (HDM) three times per week for 3 weeks using a previously established protocol (Gregory et al., 2009). Mice were then challenged using a methacholine dose response curve 24 h after the final HDM dose. Airway responsiveness was initially assessed in a whole-body plethysmograph, using Penh. At baseline and at lower doses of methacholine (12.5 mg/ml and 25 mg/ml), we found no significant difference in Penh between wild-type and *Dpp10* homozygotes (Fig. S4A); however, at the highest dose of methacholine tested (50 mg/ml), *Dpp10* homozygotes showed a significant increase in Penh (Fig. S4B) (*P*<0.05).

**Fig. 3.**
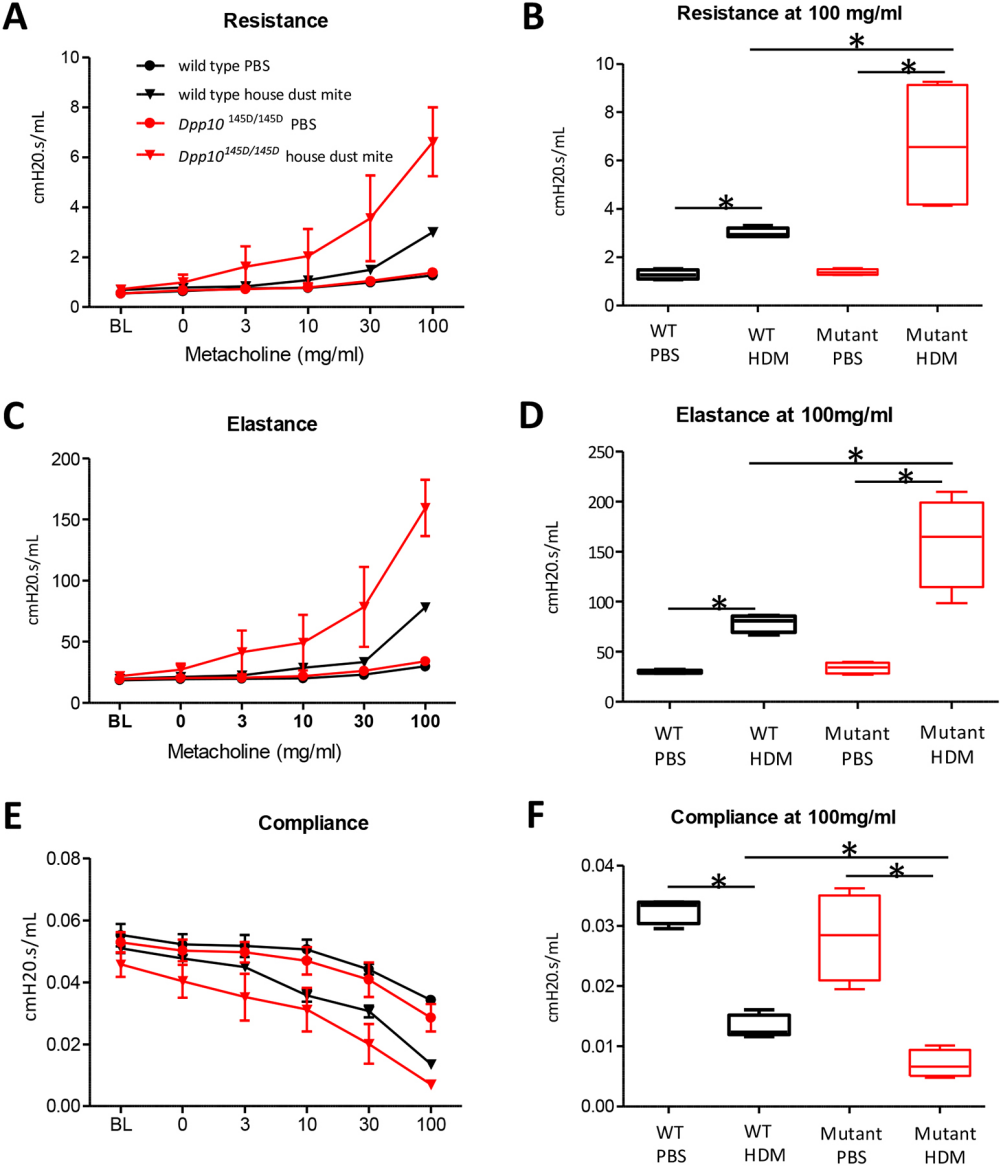
**Following HDM challenge, *Dpp10* homozygous mice show increased AHR compared to wild-type littermates.** (A-F) Lung function was measured using a FlexiVent system. The effect of methacholine on airway resistance (A,B), airway elastance (C,D) and airway compliance (E,F) is shown. Dose response curves are shown in A, C and E; data are mean±s.e.m. Airway resistance (B), airway elastance (D) and airway compliance (F) at 100 mg/ml methacholine are shown in detail. Box plots depict the median and interquartile range, whiskers show minimum and maximum values. *n*=4 mice for PBS groups, *n*=6 for HDM groups, Mann–Whitney *U*-test, *P<0.05.

Following plethysmograph experiments, which indicated increased airway resistance in *Dpp10* homozygous mice, we conducted a further HDM challenge prior to directly testing respiratory function using the forced oscillation technique (FlexiVent). *Dpp10* HDM-treated mutant mice showed significantly increased airway resistance in response to methacholine challenge (Fig. 3A) at 100 mg/ml compared to wild-type HDM treated mice (*P*<0.05, Fig. 3B). *Dpp10* HDM-treated mutant mice also showed significantly increased elastance (Fig. 3C,D, *P*<0.05) and decreased compliance (Fig. 3E,F, *P*<0.05) in response to methacholine challenge at 100 mg/ml compared to wild-type HDM-treated mice.

**Fig. 4.**
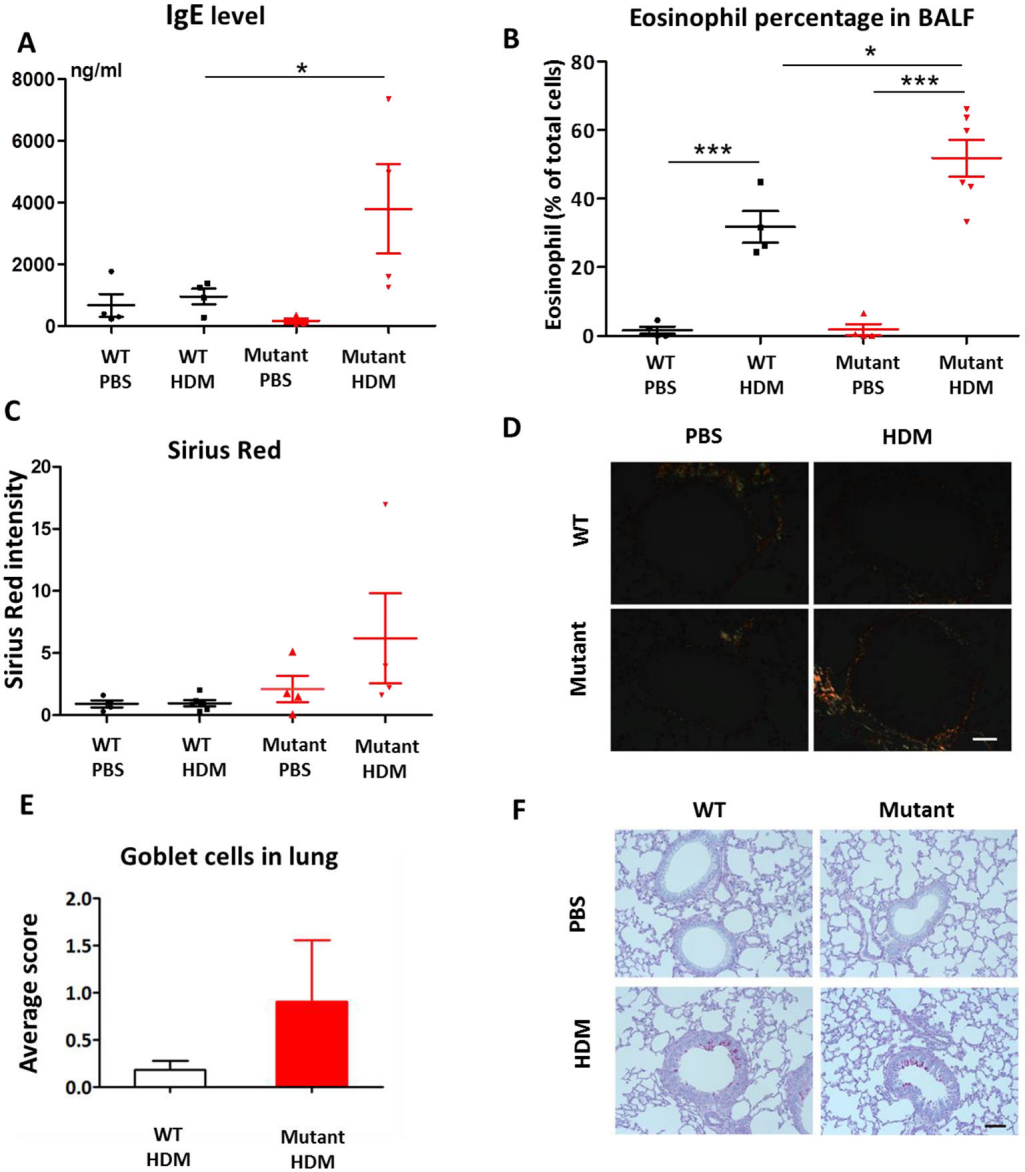
***Dpp10* homozygous mice develop allergic inflammation in the lungs after HDM challenge.** (A,B) Exposure of *Dpp10* homozygotes to HDM resulted in enhanced serum IgE levels (A) and elevated eosinophil counts in bronchoalveolar lavage fluid (BALF) (B) compared to wild-type littermates. (C-F) *Dpp10* homozygotes showed a nonsignificant trend towards increased peribronchiolar collagen (C,D) and goblet cell numbers compared to wild-type littermates (E,F). Data are mean±s.e.m.; *n*=4 mice for all groups of IgE (A); *n*=4 for WT PBS, WT HDM and *Dpp10* PBS groups, *n*=6 for *Dpp10* HDM group (B); *n*=4 for WT PBS, *Dpp10* PBS and *Dpp10* HDM groups, *n*=6 for WT HDM group (C); *n*=3 for WT and *Dpp10* HDM groups (E). Mann–Whitney *U*-test, *P<0.05, ***P<0.001. Scale bars: 12.5 µM in D, 25 µM in F.

To analyse the response of *Dpp10* homozygous mice to allergen challenge further, we assessed a number of hallmarks of allergic airways disease. IgE levels in serum of HDM-treated *Dpp10^145D^* mice were significantly higher compared to those in wild-type HDM-treated littermates (Fig. 4A, *P*<0.05). We also found an increased percentage of eosinophils in bronchoalveolar lavage fluid (BALF) of *Dpp10^145D/145D^* HDM-treated mice compared to that in the wild-type HDM group (Fig. 4B, *P*<0.05). Quantification of collagen thickness around airways showed a trend towards increased thickness in *Dpp10^145D^* homozygotes; however, this was not statistically significant (Fig. 4C,D). We did not observe any noticeable difference in goblet cell numbers across all groups of mice (Fig. 4E,F).

### DPP10 protein is present in human asthmatic airway epithelial cells and modulation of *DPP10* affects key inflammatory mediators

**Fig. 5.**
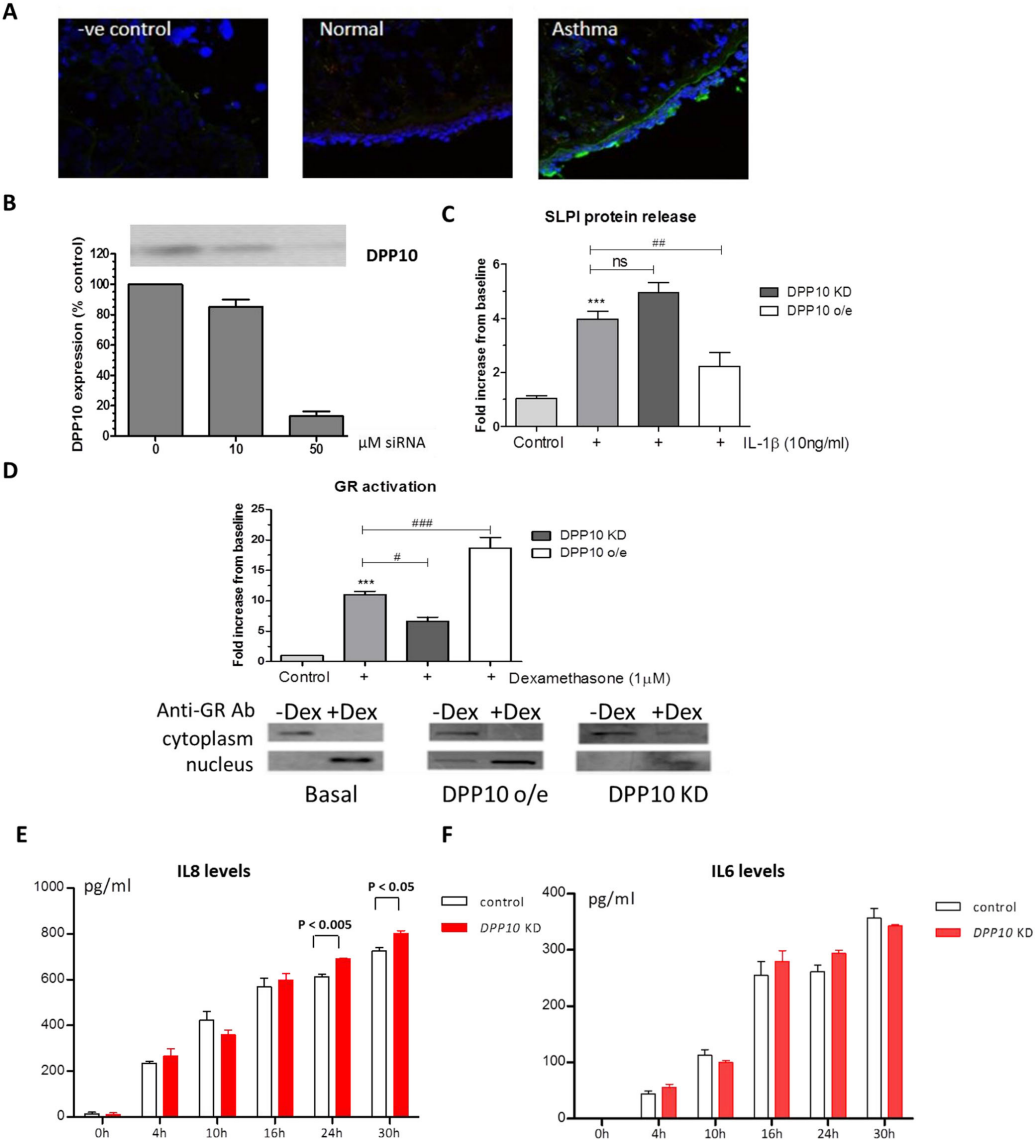
**DPP10 is increased in human asthmatic airways and modulates release of inflammatory mediators from human airway epithelial cells.** (A) Representative immunohistochemical staining of bronchial biopsies from asthmatic and normal healthy subjects. Results are representative of samples from three subjects. (B) A concentration-dependent reduction in DPP10 protein expression was observed after 24 h in BEAS-2B cells following siRNA knockdown. (C) DPP10 overexpression suppresses IL1β-induced SLPI release from BEAS-2B cells, whereas DPP10 knockdown results in an increase in SLPI release. (D) *DPP10* knockdown also attenuates the ability of the glucocorticoid receptor (GR) to be activated by dexamethasone, whereas DPP10 overexpression enhanced GR DNA binding. The lower panel shows representative western blots of GR translation. (E) IL8 levels in supernatants from *DPP10* knockdown NHBE cells and control cells after 0-30 h stimulation of IL1β. (F) IL6 levels in supernatants from *DPP10* knockdown and control NHBE cells after 0-30 h stimulation of IL1β. Data are mean±s.e.m. from three or four independent experiments. Student's *t*-test, ***P<0.001 versus control; ^###^*P*<0.001, ^##^*P*<0.01 and ^#^*P*<0.05 versus stimulated samples; ns, nonsignificant.

In order to investigate the possible functional roles of DPP10 in human lungs, we also investigated DPP10 expression in asthma patients. In agreement with our findings in mouse, we found very little DPP10 protein in the bronchial airway epithelium of healthy subjects. By contrast, expression was enhanced in the epithelium of asthmatic patients (Fig. 5A). As with the murine data, DPP10 was only seen in some epithelial cells.

Data from the murine model indicated a potential protective role of DPP10 in the airways so we investigated the effects of DPP10 knockdown and overexpression in human bronchial epithelial cells (BEAS-2B). *DPP10* small interfering RNA (siRNA) effectively silenced the expression of DPP10 (Fig. 5B). Knockdown of DPP10 enhanced IL1β-induced leukocyte proteinase inhibitor (SLPI) release from BEAS-2B cells, although this did not reach significance (Fig. 5C). By contrast, overexpression of DPP10 resulted in a significant reduction in IL1β-induced SLPI release (Fig. 5C). As the functional effect of DPP10 *in vivo* suggested a possible effect on endogenous anti-inflammatory corticosteroid production, we examined the effect of *DPP10* modulation on the glucocorticoid receptor (GR). DPP10 knockdown attenuated the ability of GR to translocate to the nucleus and bind to DNA, whereas DPP10 overexpression significantly enhanced GR activation even without dexamethasone treatment (Fig. 5D). These data were confirmed by western blot analysis of GR nuclear translocation in BEAS-2B cells (Fig. 5D, lower panels).

To further investigate whether DPP10 could modulate cytokine release, we also conducted siRNA-mediated knockdown of DPP10 in primary epithelial cells [normal human bronchial epithelial (NHBE) cells]. Knockdown with 150 nM *DPP10* siRNA for 48 h resulted in a 66% reduction in *DPP10* transcript levels, compared to the control. Following siRNA knockdown, NHBE cells were stimulated with IL1β and the levels of IL8 (Fig. 5E) and IL6 (Fig. 5F) released from control and DPP10 knockdown cells were measured at 0, 4, 10, 16, 24 and 30 h following stimulation. *DPP10* knockdown increased the level of IL8 and IL6 released from NHBE cells at 24 and 30 h (the increase was statistically significant for IL8 but not IL6). These results are consistent with the idea that Dpp10 plays a protective role in asthma.

## DISCUSSION

Using a *Dpp10* point mutant, with a valine to aspartic acid change in the β-propeller region, we demonstrated that homozygous mutant mice exhibit greater AHR following HDM challenge than wild-type animals. We also find that DPP10 protein is present in airway epithelial cells and Dpp10 immunostaining is increased in mice following HDM challenge; this corresponds with clinical findings where asthmatic patients show increased DPP10 in lungs compared to controls. Modulation of DPP10 in BEAS-2B and NHBE cells resulted in altered levels of cytokine release following IL1β stimulation, as well as modified glucocorticoid function. These results provide biological evidence to support previous findings from human genetic studies indicating that *DPP10* is an asthma susceptibility gene.

Positional cloning and GWAS have identified several genes that are associated with asthma. Understanding the functions of these genes is an important next step to shed light on the aetiology of asthma. This is particularly important for genes like *Dpp10*, where very little is known about their biological function in the lungs (Zhang et al., 2012). The association of *DPP10* with asthma has been confirmed across several different ethnic populations (Wu et al., 2010; Blakey et al., 2009; Zhou et al., 2009; Gao et al., 2010; Mathias et al., 2010). In addition, recent studies have shown that *DPP10* genetic variants affect lung function decline in ageing (Poon et al., 2014), and have also been associated with aspirin-exacerbated respiratory disease (Kim et al., 2015). DPP10 is a potassium channel-associated protein (Chen et al., 2014; Kitazawa et al., 2015), but unlike other DPP family members, mammalian DPP10 lacks enzymatic activity and is unable to cleave terminal dipeptides from asthma-related cytokines and chemokines (Allen et al., 2003). Interestingly, however, in *Drosophila*, DPP10 both acts as an ion channel substrate and has peptidase activity (Shiina et al., 2016).

DPP proteins contain a β-propeller domain, which regulates substrate access to an α/β-hydrolase catalytic domain. Most interactions of DPP10 with other proteins are likely to occur on the β-propeller domain so it is significant that the mutation in *Dpp10^145D^* is in this domain (Chen et al., 2014). In the brain, DPP10 malfunction is associated with neurodegenerative conditions such as Alzheimer’s disease and frontotemporal lobe dementia. Moreover, *DPP10* variants in neurons have been shown to alter potassium channel-gating kinetics (Jerng et al., 2007), and additional studies have shown that DPP10 modulates the electrophysiological properties, cell-surface expression and subcellular localization of voltage-gated potassium channels (Bezerra et al., 2015). Given the location of the mutation in the *Dpp10^145D^* mouse line, it is tempting to speculate that ion channel-gating kinetics could also be altered in the lungs of these mice.

Like human *DPP10*, mouse *Dpp10* has 26 exons and multiple splice variants, which retain the transmembrane domain and β-propeller domains. Mouse ENU mutagenesis has proven to be a powerful tool for studies of human diseases, particularly prior to the discovery of Crispr/Cas9 gene editing techniques. Through backcrossing, mice harbouring the selected mutation on a congenic background can be obtained (Keays et al., 2006). In the current experiment, we identified one nonsynonymous mutation, resulting in an amino acid change from valine to aspartic acid in the β-propeller domain of *Dpp10*. Careful comparison of embryonic *Dpp10* homozygotes and wild-type littermates did not reveal any visible phenotype, indicating that the *Dpp10^145D^* mutation does not affect the development of mouse lungs.

In this study, we found that mouse Dpp10 is localized to the airway epithelium, and more Dpp10 is visible in airways, after HDM treatment. This finding is consistent with a previous report showing Dpp10 protein in the airway epithelium of rat lungs (Schade et al., 2008). Comparison of airways stained for Dpp10 and CC10, a marker of club cells, indicates that not all club cells are positive for Dpp10, because CC10-stained cells are more prevalent than Dpp10-positive cells. Our data indicate that at least some CC10-positive cells are also positive for Dpp10. However, further investigation with additional cell type-specific markers will be required to determine which subtype(s) of airway epithelial cells express Dpp10, e.g. basal cells, ciliated cells etc. Comparison of the subcellular pattern of CC10 staining with that of DPP10 revealed differences in the localization of these two proteins. Moreover, the localization of Dpp10 protein is different in airways of wild-type and *Dpp10^145D^*; there are significantly fewer cells with Dpp10 at the apical surface in mutant lungs. These observations indicate that loss of function in the *Dpp10^145D^* mice is likely to result from altered protein function rather than lack of protein. This is a frequent consequence of point mutations in both mouse models and in the human population (Poobalasingam et al., 2017; Piel et al., 2017).

HDM is one of the commonest aeroallergens worldwide and up to 85% of asthmatics are typically HDM allergic. Inhalation of HDM by naïve mice results in lung function changes, including increased resistance and reduced compliance, as well as recruitment of inflammatory leukocytes (Gregory et al., 2009; Johnson et al., 2004). HDM challenge initiates a Th2-polarized response with T2 helper cytokines being produced in both BALF and lungs of mice, including IL4, IL5 and IL13, and typically an influx of eosinophils and increase in IgE levels. This cytokine response is important, driving the development of airway remodelling, which occurs after the Th2 cytokine response, and altered lung function (Gregory et al., 2009; Johnson et al., 2007).

Our results show that after HDM challenge, *Dpp10* homozygotes display significantly increased AHR upon methacholine challenge compared to wild-type littermates. Specifically, resistance and elastance are increased and compliance is reduced. Following HDM treatment, serum IgE levels and eosinophil counts in BALF were significantly higher in *Dpp10* mutants after HDM treatment. Although collagen thickness and goblet cells were not significantly increased, it is known that these hallmarks of airway remodelling only become significantly altered after a longer and more frequent HDM dosing regimen than the one used here (Gregory et al., 2009, 2010; Johnson et al., 2004).

In asthmatics, exposure to the allergen HDM triggers the release of cytokines as a result of mite-derived protease activity (Seto et al., 2009). Anti-proteases such as SLPI can inhibit this cytokine release. *SLPI* is an anti-inflammatory gene induced by glucocorticoid in human epithelial cells and low SLPI has been associated with severe asthma in mice and humans (Sallenave et al., 1994; Raundhal et al., 2015). In this study, we showed that DPP10 overexpression in BEAS-2B cells results in lower levels of SLPI release following cytokine stimulation and enhanced corticosteroid activity. In combination with our finding that *DPP10* knockdown in primary human bronchial epithelial cells leads to increased IL8 and IL6 release, these data demonstrate that alterations in *DPP10* levels can modify the inflammatory response in lung epithelial cells.

In summary, we have established a congenic mouse line harbouring a mutation in the β-propeller domain of DPP10. Our data provide functional experimental evidence supporting previous human genetic data indicating that *DPP10* is an asthma susceptibility gene. Results from both murine and human *in vivo* and *in vitro* analyses suggest that DPP10 might play a protective role in asthma. The novel *Dpp10* mouse mutant reported here provides a means to investigate the functional roles of *Dpp10 in vivo*, for the first time. Understanding how the mutant *Dpp10* potassium voltage-gated ion channel complex is impacted by the DPP10^145D^ mutation, and how this affects other inflammatory associated factors such as TGFβ and IL33, will be important future studies.

## MATERIALS AND METHODS

### Full-length cDNA of *Dpp10* and exon sequencing

A mouse cDNA clone (BE862767) was obtained and extended via its 5′ and 3′ ends to a full-length cDNA using rapid amplification of cDNA ends (RACE) (Clontech). The primary transcript encodes a novel 2370 bp open reading frame (ORF) with a predicted peptide sequence of 789 residues. To identify mutations in *Dpp10*, we screened four exons of *Dpp10* (2, 5, 6 and 7) in 3840 mutagenized BALB/C DNA samples from the UK MRC Harwell ENU archive by sequencing the PCR products. Exon 2 encodes the transmembrane amino acids and exon 5, 6, and 7 encode the first and second β-propellers of the peptide. PCR primer sequences are listed in Table S1. PCR conditions were as follows: 35 cycles consisting of (i) 60 s at 94°C, followed by (ii) 60 s at 50-60°C and then (iii) 30 s at 72°C. PCR products from all 3840 DNA samples were purified with Millipore purification plates and sequenced using an ABI 3700 sequence machine. Sequence traits were aligned and DNA samples carrying mutations were identified. The mutation predicted to most likely affect function was used to establish the DPP10 mouse line.

### Mouse line recovery and maintenance

Mice were housed and maintained in accordance with the rules and regulations of the UK Animals (Scientific Procedures) Act 1986, and the Harwell Animal Welfare and Ethical Review Body (AWERB). Mice were maintained in specific pathogen-free conditions and provided food and water *ad libitum*. Sperm containing the *Dpp10* mutation identified above was recovered from the parallel Harwell sperm archive and used in IVF with C3H females to obtain live mice carrying this *Dpp10* mutation. Progeny born following IVF were genotyped using a diagnostic restriction enzyme digest followed by PCR as follows: (i) 60 s at 94°C, (ii) 60 s at 55°C and (iii) 30 s at 72°C for 32 cycles. PCR products were digested with the HpyCH4IV enzyme (2 U for 3 h), then run on a 2% agarose gel to determine the size of products. The mutant *Dpp10* band was 194 bp and wild-type DNA was cut into two fragments of 127 bp and 62 bp. We subsequently established a congenic *Dpp10* line by backcrossing these mice to CH3 for 10 generations.

### Histology and immunostaining of lung tissues

Adult human lung tissue was obtained from the Biobank of the Respiratory Biomedical Research Unit (BRU), Royal Brompton and Harefield NHS Foundation Trust. The study was approved by the Trust's Ethics Committee (ethics reference number 10/H0504/9). Informed written consent was obtained from all study subjects. Control samples were obtained from healthy, nonasthmatic volunteers. Four micrometre parafﬁn sections or 10 µm cryosections were stained with Haematoxylin and Eosin (H&E), Mauritius Scarlet Blue (MSB) to identify collagen, or immunostained using previously established protocols (Yates et al., 2010). Lung tissue processing and immunohistochemistry were performed as previously described (Varani et al., 2006). Antibodies used were as follows: anti-aquaporin-5, 1:400, Santa Cruz Biotechnology; anti-CC-10, 1:500, Santa Cruz Biotechnology; anti-pro-SP-C, 1:1000, Chemicon; anti-alpha SMA, 1:1000, Lab Vision. Incubations were overnight at 4°C. For CC10 immunostaining, antigen retrieval using citrate buffer (pH 6) was required to unmask antigens prior to immunodetection. Antibodies were detected with either a universal (alpha SMA, pro-SP-C) or goat (CC10, aquaporin-5) ABC elite staining kit (Vector Labs). Rabbit anti-DPP10 was obtained from Santa Cruz Biotechnology (sc-174156) or Aviva Systems Biology (OAAB05596) and used at 1:100 dilution. Controls, where the primary antibody was omitted, were included in each immunostaining experiment.

### Quantifying the percentage of airway cells with apical Dpp10 immunostaining

The total number of cells in individual airways was determined by counting the number of Haematoxylin-stained nuclei. The percentage of cells with apical Dpp10 staining in each airway was determined by expressing the number of cells with brown staining visible at the apical surface of cells (i.e. immediately adjacent to the lumen) as a percentage of total airway cells. Eight airways from *n*=3 mice per group were analysed.

### Allergen sensitization

Eight- to 10-week-old mice (no more than 1 week difference in age within any one experiment) were dosed with purified HDM extract (batch number 7500, Greer Laboratories, NC, USA; 21.26 µg derP/mg protein, 13.55 endotoxin U/mg) intranasally (1 µg/µl in 25 µl PBS three times/week for 3 weeks; littermate control mice received PBS only). Lung function testing was carried out 24 h after the last dose.

### Measurements of AHR

Unrestrained conscious mice (male and female) were placed into a plethysmograph chamber (Buxco Europe, Hampshire, UK). Airway resistance was measured as Penh (enhanced pause) (Solberg et al., 2006). Baseline Penh was measured for 5 min, after which time an aerosol of methacholine (Sigma-Aldrich, Dorset, UK) was given and Penh was again measured for 5 min. The change in Penh was calculated between baseline and each subsequent methacholine dose.

Respiratory mechanics were also directly measured using the forced oscillation technique (FlexiVent system, SCIREQ) in anaesthetized, ventilated female mice 24 h postchallenge. Following lung function measurements, serum, BALF and lung tissue samples were collected and processed based on methods by Hamelmann et al. (1997). BALF was collected using 3×0.4 ml sterile PBS and centrifuged. The supernatant was stored for further analysis, and the cell pellet was re-suspended in 0.5 ml RMPI, supplemented with 10% foetal bovine serum, penicillin and streptomycin. Then, 50,000 cells were spun at 31 ***g*** for 4 min using a Cytospin3 (Shandon) and fixed in methanol for 5 min. Cells were stained using Wright-Giemsa stain (Sigma-Aldrich) following the manufacturer's instructions. Eosinophil percentages were obtained by determining the number of eosinophils in a sample of 300 cells per mouse. Left lung lobes were inflated and fixed for histology as detailed above.

### Quantification of peribronchiolar collagen and goblet cell counts in lung tissue samples

To quantify collagen around the airways, 4 µm wax sections were stained with Sirius Red. Digital images were obtained from airways that were similar in size using a 40× lens under polarized light. All images were obtained in one sitting to minimize any variability. For each image, a threshold was used so only the Sirius Red staining was highlighted, and the integrated density of staining around each airway was determined. Image analysis was performed using Fiji software. For each mouse, four airways were measured and the mean average was calculated.

Goblet cells counts were obtained using the protocol described in Townsend et al. (2000). Periodic Acid-Schiff (PAS)-stained goblet cells in airway epithelium were counted in 4 µm wax lung sections. Counting was undertaken blind using a numerical scoring system (0, 5% goblet cells; 1, 5 to 25%; 2, 25 to 50%; 3, 50 to 75%; 4, >75%). The sum of airway scores from each lung was divided by the number of airways examined (20-50 per mouse) and expressed as mucus cell score in arbitrary units (U).

### Cell culture of human bronchial airway epithelial cells

Bronchial epithelial transformed cells (BEAS-2B) were purchased from LGC standards (Teddington, UK). No contamination was found in the cell stock. The cells were cultured in keratinocyte media, supplemented with recombinant human epithelial growth factor (rhEGF) and bovine pituitary extract (BPE) (all Gibco, Paisley, UK) as previously described (Khan et al., 2014). Following overnight culture in minimal media to enable synchronization, cells were stimulated with IL1β (10 ng/ml, Sigma-Aldrich) or dexamethasone (Sigma-Aldrich) and proteins isolated at 2 h or 24 h for analysis of supernatants by enzyme-linked immunosorbent assay (ELISA). Proteins were extracted from cells using a nuclear extraction kit (Active Motif, Rixensaart, Belgium) following the manufacturer’s instructions.

### Knockdown and overexpression of DPP10 in BEAS-2B cells

*DPP10* gene expression was knocked down using 50 nM ON-TARGET plus SMART pool siRNA (Thermo-Scientific Dharmacon, CO, USA) against the *DPP10* gene as described previously (Khan et al., 2014). Silencer select siRNA (Ambion, TX, USA) was used as a control for transfection. The DPP10 clone 11 (Allen et al., 2003) was overexpressed using jetPEI (Polyplus-transfection, Illkirch, France) according to the manufacturer's instructions.

### Knockdown of DPP10 in NHBE cells

Primary NHBE cells were obtained from Lonza and no contamination was found in the cell stock. The cells were cultured to a maximum of five passages to limit variable responses in bronchial epithelial medium (hAEC Culture Medium, Epithelix). Cells were seeded into 24-well plates (Corning Costar Corp.) at 4×10^4^ cells/well to reach 40-60% confluence on the day of transfection. RNA interference (RNAi) was carried out using 150 nM ON-TARGETplus SMARTpool (Dharmacon Research Inc., Lafayette, CO, USA) or ON-TARGETplus Non-targeting Pool negative control. RNAi complexes were added to the cells, which were placed in a CO_2_ incubator at 37°C for 48 h. Following transfection, the culture medium was changed and then stimulated with 1 ng/ml IL1β. Cell supernatants were collected at different time points poststimulation (0, 4, 10, 16, 24 and 30 h) for cytokine measurement. Quantitative PCR primer sequences were as follows: *DPP10* forward primer 5′-GTGAAGGTCCAAGGGTC-3′; reverse primer 5′-CTGGCTTTCCTATCTTCTTC-3′. *GAPDH* forward primer 5′-TCAAGAAGGTGGTGGTGAAGCAG-3′; reverse primer 5′-CGCTGTTGAAGTCAGAGGAG-3′.

### ELISA

Levels of secretory leukocyte protease inhibitor (SLPI) in the supernatant were measured by ELISA, following the manufacturer's instructions (R&D Systems, Minneapolis, MN, USA), 24 h after cell stimulation. Human IL6 and IL8 were measured with an ELISA DuoSet (R&D Systems Europe, Abingdon, UK), according to the manufacturer's instructions. Mouse serum IgE levels were measured by ELISA using purified anti-mouse IgE capture antibody (553413, BD Pharmingen), according to the manufacturer's instructions.

### Western blotting

Western blotting was performed as described previously (Khan et al., 2014) using an anti-GR antibody (H-300, Santa Cruz Biotechnology) at 1:1000 dilution, and visualized using Luminata™ Forte solution (Millipore, Billarica, MA, USA) with exposure to X-ray film (Fisher Scientific, Loughborough, UK).

### Statistical analysis

Graphs were generated using GraphPad Prism software (GraphPad version 5.0, La Jolla, CA, USA) or Microsoft Excel. Where appropriate, sample sizes were determined using power calculations based on previous experimental data. Data are expressed as mean±s.e.m. Data on resistance, elastance and compliance at 100 mg/ml methacholine and ΔPenh at 50 mg/ml methacoline are expressed as box and whisker plots showing the median, interquartile range, and minimum and maximum values. For HDM challenge experiments, statistical significance of HDM-treated versus PBS control groups in wild-type and *Dpp10^145D^* mice was determined by a two-tailed *P* value using the Mann–Whitney *U*-test when comparing two groups only. For knockdown experiments in human cells, graphs show mean±s.e.m. of SLPI, IL6 and IL8 for *DPP10* knockdown cells and control cells. Differences between groups were tested using a two-tailed Student's *t*-test. *P*<0.05 was considered significant for all statistical analyses.

## Supplementary Material

Supplementary information
