# Supplementary material for: Manipulation of dipeptidylpeptidase 10 in mouse and human in vivo and in vitro models indicates a protective role in asthma
Source: Dis Model Mech. 2018 Jan 1;11(1):dmm031369. doi: 10.1242/dmm.031369 (PMC5818078; doi:10.1242/dmm.031369)
Supplement: Supplementary information [file dmm-11-031369-s1.pdf]

FIRST PERSON

First person – Youming Zhang

First Person is a series of interviews with the first authors of a selection of papers published in Disease Models & Mechanisms, helping early-career researchers promote themselves alongside their papers. Youming Zhang is first author on 'Manipulation of dipeptidylpeptidase 10 in mouse and human *in vivo* and *in vitro* models indicates a protective role in asthma', published in DMM. Youming is a lecturer at the National Heart and Lung Institute, Imperial College London, UK, investigating the functional roles of novel genes in respiratory diseases.

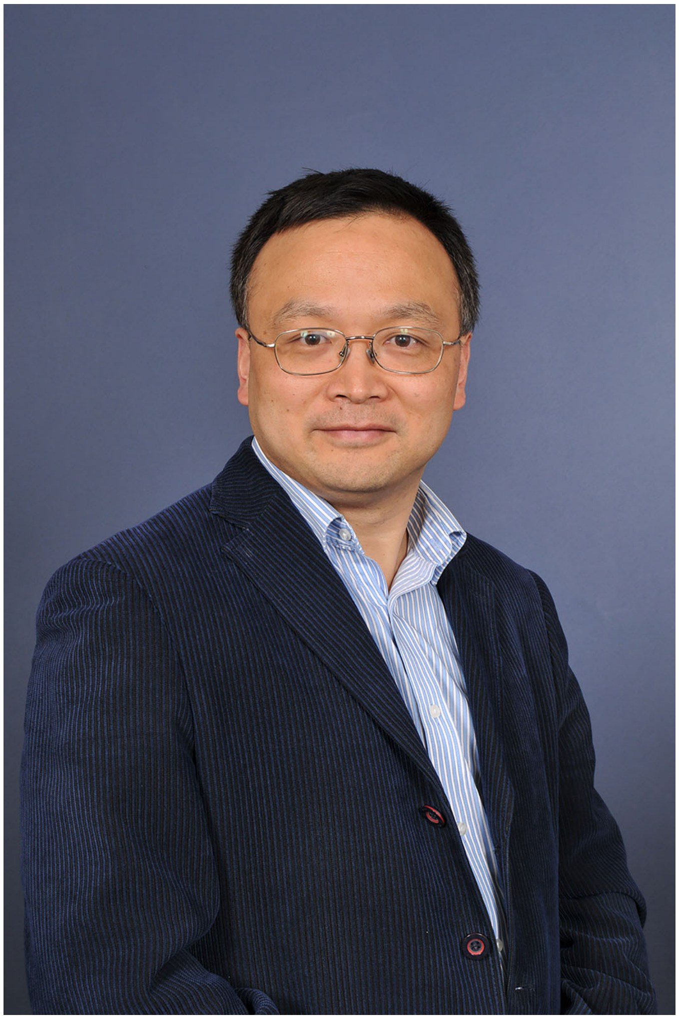

Youming Zhang

How would you explain the main findings of your paper to non-scientific family and friends?

Asthma is a highly prevalent chronic respiratory disease worldwide. The disease is caused by a combination of genetic factors and environmental factors. *DPP10* is a gene identified by genetic studies to have association with asthma in many populations, but we still do not know how the gene works in asthma patients' lungs. Therefore, we established a mouse model of mutant *DPP10* after screening the DNA of 3920 mice that were treated by a chemical mutagenesis agent ENU. We examined the mutant mouse lungs after the mice were challenged by a common allergen, the house dust mite. The mutant mice showed some human asthma characteristics in their lungs. We also examined the DPP10 in human lung cells and found it had a protective role in asthma pathophysiology.

What are the potential implications of these results for your field of research?

This was the first time to show the voltage-gated potassium channel protein DPP10 to have roles in regulation of IgE production, cytokine release and collagen deposition in mouse lungs. Understanding how DPP10 regulates the processes will bring new insights into the mechanism of the airway disease.

> "Luck may not always exist, but hard working, detailed observation, consistence and collaboration are the important keys for success."

What are the main advantages and drawbacks of the model system you have used as it relates to the disease you are investigating?

The beauty of this mouse model is to provide a unique tool to understand the role of transmembrane protein DPP10. The mouse model just had one single amino acid change in a conserved region of DPP10 and it had brought dramatic phenomena in mouse lungs after the mice were challenged by house dust mite. This model is suitable for studying the interactions between DPP10 and other molecules in the mechanism of asthma. It is also a useful model for studying neurological disorders that are related by voltage-gated potassium channels. The drawback of this model is that the phenotypes from the mutant mice will require a lot of effort and time to investigate and observe.

What has surprised you the most while conducting your research?

I conducted the experiments and thought the location of the mutant DPP10 protein would express on the same position in the cell membrane as the wild type expressed. In reality we found this mutant protein expressed much less in apical surfaces of airway epithelial cells of the mutant mice. The change of the location from apical surface to basal surface of DPP10 was very interesting and it might hold a key to understanding DPP10's role in epithelial cells.

Youming Zhang's contact details: National Heart and Lung Institute, Imperial College London, UK.
E-mail: y.zhang@imperial.ac.uk

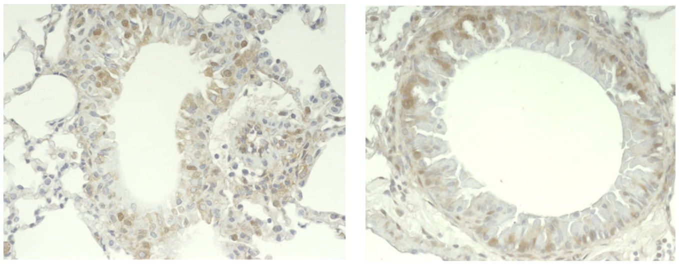

DPP10 location in wild-type (left) and mutant (right) mouse lungs.

Describe what you think is the most significant challenge impacting your research at this time and how will this be addressed over the next 10 years?

Understanding how genetic and environmental factors work together to cause complicated diseases such as asthma is the major challenging in my research at this time. In 10 years' time, with the development of genomics and other techniques, many questions will be answered. We will know how the diseases are caused and how to prevent and treat the diseases effectively.

What changes do you think could improve the professional lives of early-career scientists?

For early-career scientists, it is always right to have persistence in the pursuit of the research. Luck may not always exist, but hard working, detailed observation, consistence and collaboration are the important keys for success.

What's next for you?

I will continue to work on the dissecting functional roles of the genes underlying asthma. I am particularly interested in the pathways the genes involve in epithelial cells during allergic response. New insight of the pathways will bring new potential therapeutic means to the airway disease.

Reference

Zhang, Y., Poobalasingam, T., Yates, L. L., Walker, S. A., Taylor, M. S., Chessum, L., Harrison, J., Tsaprouni, L., Adcock, I. M., Lloyd, C. M., Cookson, W. O., Moffatt, M. F. and Dean, C. H. (2018). Manipulation of dipeptidylpeptidase 10 in mouse and human *in vivo* and *in vitro* models indicates a protective role in asthma. *Dis. Model. Mech.* **11**, doi:10.1242/dmm.031369M.
